# Supplementary material for: Epidemiological baseline of Brucella spp. in South African wildlife
Source: PLoS Negl Trop Dis. 2025 Dec 11;19(12):e0013754. doi: 10.1371/journal.pntd.0013754 (PMC12716795; doi:10.1371/journal.pntd.0013754)
Supplement: S3 File — (ZIP) [file pntd.0013754.s003.zip › S3_amos_pcr.docx]

Quick SOP nr.16 – 06/02/2025

*Brucella* spp. AMOS touchdown cPCR

Compiled by: Carlo Andrea Cossu

This protocol describes a conventional PCR that is able to detect and differentiate four *Brucella* species namely *B. abortus, B. melitensis, B. ovis* and *B. suis*. The assay targets the insertion element IS711, which occurrs in different position of *Brucella* species genome. Primer details are displayed in Table [**1**](#PrimersTable).

**Table** **1:** Primer details

| **Target gene** | **Primers** | **Nucleotide.sequence (5’-3’)** | **Amplicon length (bp)** | **Reference** |
| --- | --- | --- | --- | --- |
| IS711 | *B. abortus* | GACGAACGGAATTTTTCCAATCCC | 498 | [Bricker & Halling, 1994](#Bricker_1994) |
|  | *B. melitensis* | AAATCGCGTCCTTGCTGGTCTGA | 731 |  |
|  | *B. ovis* | CGGGTTCTGGCACCATCGTCG | 976 |  |
|  | *B. suis* | GCGCGGTTTTCTGAAGGTTCAGG | 285 |  |
|  | IS711 reverse primer | TGCCGATCACTTAAGGGCCTTCAT | - |  |

PCR mixture is prepared as shown in Table [**2**](#MixTable). The reaction is performed in a thermocycler as displayed in Table [**3**](#ThermocyclerTable). PCR products are loaded on 2% agarose gels with 3% ethidium bromide and separated on gel electrophoresis apparatus at 120V (400mA).

For all PCR reactions, double distilled water was used as negative control, while the *B. abortus* RB51, *B. melitensis* Rev.1, *B. suis* 1330 and *B. ovis* (from clinical sample) are used as positive controls.

**Table** **2:** Details of PCR mix.

| **Component** | **Initial concentration** | **Final concentration** | **Volume x 1 (µL)** |
| --- | --- | --- | --- |
| MyTaq Red Mix | 2x | 1x | 7.5 |
| Forward primer set | 20 µM | 0.1 µM | 0.075 |
| Reverse primer set | 20 µM | 0.1 µM | 0.075 |
| dH20 | NA | | 5.35 |
| Subtotal | NA | | 13.0 |
| DNA | At least 3 ng/µl | At least 6 ng | 2.0 |
| Total | NA | | 15.0 |

**Table** **3:** Thermocycler conditions.

| **Step** | **Temperature (°C)** | **Time** | **Nr. cycles** |
| --- | --- | --- | --- |
| Initial denaturation | 95 | 2 min | 1 |
| Denaturation | 95 | 15 sec | 10 |
| Annealing | 70 to 60  (2° reduction every two cycles) | 30 sec |  |
| Elongation | 72 | 20 sec |  |
| Denaturation | 95 | 15 sec | 40 |
| Annealing | 60 | 30 sec |  |
| Elongation | 72 | 20 sec |  |
| Final elongation | 72 | 1 min | 1 |

## REFERENCES

Bricker, B. J., & Halling, S. M. (1994). Differentiation of brucella abortus bv. 1, 2, and 4, brucella melitensis, brucella ovis, and brucella suis bv. 1 by PCR. *Journal of Clinical Microbiology*, *32*, 2660–2666. <https://doi.org/10.1128/jcm.32.11.2660-2666.1994>
