# Supplementary material for: Epidemiological baseline of Brucella spp. in South African wildlife
Source: PLoS Negl Trop Dis. 2025 Dec 11;19(12):e0013754. doi: 10.1371/journal.pntd.0013754 (PMC12716795; doi:10.1371/journal.pntd.0013754)
Supplement: S3 File — (ZIP) [file pntd.0013754.s003.zip › S3_bruce_ladder.docx]

Quick SOP nr.13 – 21/03/2024

*Brucella* spp. Bruce-Ladder multiplex PCR

Compiled by: Carlo Andrea Cossu

This protocol describes a multiplex PCR that is able to detect and differentiate eight of the currently known *Brucella* species *i.e. B. abortus, B. melitensis, B. ovis, B. suis, B. canis, B. pinnipedalis, B. ceti* and *B. neotomae*. The assay is designed and interpreted on the presence/absence of eight different gene targets. Primer details are displayed in Table **[1](#PrimersTable)**.

**Table** **1:** Primer details

| **Target gene** | **Primers** | **Nucleotide.sequence (5’-3’)** | **Amplicon length (bp)** | **Reference** |
| --- | --- | --- | --- | --- |
| Glycosyltransferase (wboA) | BMEI0998f | ATC CTA TTG CCC CGA TAA GG | 1682 | [López-Goñi et al., 2008](#ref-LopezGoni2008) |
|  | BMEI0997r | GCT TCG CAT TTT CAC TGT AGC |  |  |
| Immunodominant antigen (bp26) | BMEI0535f | GCG CAT TCT TCG GTT ATG AA | 450 (1320) |  |
|  | BMEI0536r | CGC AGG CGA AAA CAG CTA TAA |  |  |
| Outer membrane protein (omp31) | BMEII0843f | TTT ACA CAG GCA ATC CAG CA | 1071 |  |
|  | BMEII0844r | GCG TCC AGT TGT TGT TGA TG |  |  |
| Polysaccharide deacetylase | BMEI1436f | ACG CAG ACG ACC TTC GGT AT | 794 |  |
|  | BMEI1435r | TTT ATC CAT CGC CCT GTC AC |  |  |
| Erythritol catabolism (eryC) | BMEII0428f | GCC GCT ATT ATG TGG ACT GG | 587 |  |
|  | BMEII0428r | AAT GAC TTC ACG GTC GTT CG |  |  |
| ABC transporter binding protein | BR0953f | GGA ACA CTA CGC CAC CTT GT | 272 |  |
|  | BR0953r | GAT GGA GCA AAC GCT GAA G |  |  |
| Ribosomal protein S12 (rpsL) | BMEI0752f | CAG GCA AAC CCT CAG AAG C | 218 |  |
|  | BMEI0752r | GAT GTG GTA ACG CAC ACC AA |  |  |
| Transcriptional regulator (CRP family) | BMEII0987f | CGC AGA CAG TGA CCA TCA AA | 152 |  |
|  | BMEII0987r | GTA TTC AGC CCC CGT TAC CT |  |  |

PCR mixture is prepared as shown in Table **[2](#MixTable)**. The reaction is performed in a thermocycler as displayed in Table **[3](#ThermocyclerTable)**. PCR products are loaded on 2% agarose gels with 3% ethidium bromide and separated on gel electrophoresis apparatus at 120V (400mA).

For all PCR reactions, double distilled water was used as negative control, while the *B. abortus* RB51, *B. melitensis* Rev.1, *B. suis* 1330, *B. ovis* (from clinical sample) and *B. canis* RM6/66 were used as positive controls.

**Table** **2:** Details of PCR mix.

| **Component** | **Initial concentration** | **Final concentration** | **Volume x 1 (µL)** |
| --- | --- | --- | --- |
| MyTaq Red Mix | 2x | 1x | 7.5 |
| Forward primer set | 6.25 µM | 0.5 µM | 1.2 |
| Reverse primer set | 6.25 µM | 0.5 µM | 1.2 |
| dH20 | NA | | 3.1 |
| Subtotal | NA | | 13.0 |
| DNA | At least 3 ng/µl | At least 6 ng | 2.0 |
| Total | NA | | 15.0 |

**Table** **3:** Thermocycler conditions.

| **Step** | **Temperature (°C)** | **Time** | **Nr. cycles** |
| --- | --- | --- | --- |
| *Initial denaturation* | 95 | 2 min | 1 |
| *Denaturation* | 95 | 30 sec | 45 |
| *Annealing* | 64 | 45 sec |  |
| *Elongation* | 72 | 3 min |  |
| *Final elongation* | 72 | 10 min | 1 |

## REFERENCES

López-Goñi, I., García-Yoldi, D., Marín, C. M., Miguel, M. J. D., Muñoz, P. M., Blasco, J. M., Jacques, I., Grayon, M., Cloeckaert, A., Ferreira, A. C., Cardoso, R., Sá, M. I. C. D., Walravens, K., Albert, D., & Garin-Bastuji, B. (2008). Evaluation of a multiplex PCR assay (bruce-ladder) for molecular typing of all brucella species, including the vaccine strains. *Journal of Clinical Microbiology*, *46*, 3484–3487. <https://doi.org/10.1128/JCM.00837-08>
