# Supplementary material for: Epidemiological baseline of Brucella spp. in South African wildlife
Source: PLoS Negl Trop Dis. 2025 Dec 11;19(12):e0013754. doi: 10.1371/journal.pntd.0013754 (PMC12716795; doi:10.1371/journal.pntd.0013754)
Supplement: S3 File — (ZIP) [file pntd.0013754.s003.zip › S3_duplex_realtime.docx]

Quick SOP nr.44 – 13/11/2024

BruAb2_BME duplex real-time PCR

Compiled by: Carlo Andrea Cossu

This protocol describes a Taqman real-time PCR that is able to detect and differentiate *Brucella abortus* and *B. melitensis*. The assay targets genetic loci that are unique of the species targeted. Primer and probe details are displayed in Table [**1**](#PrimersTable).

**Table** **1:** Primer details

| **Target gene** | **Primer/Probe name** | **Nucleotide.sequence (5’-3’)** | **Amplicon length (bp)** | **Reference** |
| --- | --- | --- | --- | --- |
| BruAb2_0168 | BruAb2_0168-F | GCACACTCACCTTCCACAACAA | 81 | [Hinic et al., 2008](#ref_Hinic2008) |
|  | BruAb2_0168-R | CCCCGTTCTGCACCAGACT |  |  |
|  | BruAb2_0168-P | FAM-TGGAACGACCTTTGCAGGCGAGATC-BHQ-1 |  |  |
| BMEII0466 | BMEII0466-F | TCGCATCGGCAGTTTCAA | 67 |  |
|  | BMEII0466-R | CCAGCTTTTGGCCTTTTCC |  |  |
|  | BMEII0466-P | VIC-CCTCGGCATGGCCCGCAA-BHQ-2 |  |  |

PCR mixture is prepared as shown in Table [**2**](#MixTable). The reaction is performed in a CFX connect or StepOne Plus as displayed in Table [**3**](#ThermocyclerTable). Any sample that reacts at a cycle threshold (Ct) < 35 is considered positive and subjected to culture on selective media.

For all PCR reactions, double distilled water was used as negative control, while the *B. abortus* RB51 and *B. melitensis* (from clinical sample) were used as positive controls.

**Table** **2:** Details of real-time PCR mix.

| **Component** | **Initial concentration** | **Final concentration** | **Volume x 1 (µL)** |
| --- | --- | --- | --- |
| QuantBio Fast Low Rox | 2x | 1x | 12,5 |
| Primers mix | 20 µM | 0.3 µM | 0,375 |
| *B. abortus* probe | 20 µM | 0.2 µM | 0,25 |
| *B. melitensis* probe | 20 µM | 0.2 µM | 0,25 |
| dH20 | NA | | 9,125 |
| Subtotal | NA | | 22,5 |
| DNA | 5-20 ng/µl | 10-50 ng | 2,5 |
| Total | NA | | 25 |

**Table** **3:** Thermocycler conditions.

| **Step** | **Temperature (°C)** | **Time** | **Nr. cycles** |
| --- | --- | --- | --- |
| *Initial denaturation* | 95 | 3 min | 1 |
| *Denaturation* | 95 | 30 sec | 40 |
| *Annealing* | 60 | 30 sec |  |
| *Elongation* | 72 | 30 sec |  |

## REFERENCES

Hinić, V., Brodard, I., Thomann, A., Cvetnić, Ž., Makaya, P. V., Frey, J., & Abril, C. (2008). Novel identification and differentiation of Brucella melitensis, B. abortus, B. suis, B. ovis, B. canis, and B. neotomae suitable for both conventional and real-time PCR systems. *Journal of microbiological methods*, *75*(2), 375-378.
