# Supplementary material for: Epidemiological baseline of Brucella spp. in South African wildlife
Source: PLoS Negl Trop Dis. 2025 Dec 11;19(12):e0013754. doi: 10.1371/journal.pntd.0013754 (PMC12716795; doi:10.1371/journal.pntd.0013754)
Supplement: S3 File — (ZIP) [file pntd.0013754.s003.zip › S3_ITS_pcr.docx]

Quick SOP nr.43 – 10/10/2024

*Brucella* spp. ITS Touchdown cPCR

Compiled by: Carlo Andrea Cossu

This protocol describes a conventional PCR that is able to detect all bacterial species belonging to the genus *Brucella*. The assay targets the 16S-23S rDNA internal transcribed spacer (ITS). Primer details are displayed in Table [**1**](#PrimersTable).

**Table** **1:** Primer details

| **Target gene** | **Primers** | **Nucleotide.sequence (5’-3’)** | **Amplicon length (bp)** | **Reference** |
| --- | --- | --- | --- | --- |
| 23S-16S ITS rDNA | ITS66 | ACATAGATCGCAGGCCAGTCA | 214 | Keid et al., 1997 |
|  | ITS279 | AGATACCGACGCAAACGCTAC |  |  |

PCR mixture is prepared as shown in Table [**2**](#MixTable). The reaction is performed in a thermocycler as displayed in Table [**3**](#ThermocyclerTable). PCR products are loaded on 1.5% agarose gels with 3% ethidium bromide and separated on gel electrophoresis apparatus at 120V (400mA).

For all PCR reactions, double distilled water was used as negative control, while the *B. abortus* RB51, *B. melitensis* Rev.1, *B. suis* 1330, *B. ovis* (from clinical sample) and *B. canis* RM6/66 were used as positive controls.

**Table** **2:** Details of PCR mix.

| **Component** | **Initial concentration** | **Final concentration** | **Volume x 1 (µL)** |
| --- | --- | --- | --- |
| MyTaq Red Mix | 2x | 1x | 12.5 |
| Forward primer | 20 µM | 0.3 µM | 0.375 |
| Reverse primer | 20 µM | 0.3 µM | 0.375 |
| dH20 | NA | | 11.25 |
| Subtotal | NA | | 24.5 |
| DNA | At least 3 ng/µl | At least 6 ng | 0.5 |
| Total | NA | | 25.0 |

**Table** **3:** Thermocycler conditions.

| **Step** | **Temperature (°C)** | **Time** | **Nr. cycles** |
| --- | --- | --- | --- |
| *Initial denaturation* | 95 | 2 min | 1 |
| *Denaturation* | 95 | 15 sec | 10 |
| *Annealing* | 72 to 62  (2° reduction every two cycles) | 30 sec |  |
| *Elongation* | 72 | 20 sec |  |
| *Denaturation* | 95 | 15 sec | 40 |
| *Annealing* | 62 | 30 sec |  |
| *Elongation* | 72 | 20 sec |  |
| *Final elongation* | 72 | 1 min | 1 |

## REFERENCES

Keid, L. B., Soares, R. M., Vasconcellos, S. A., Chiebao, D. P., Salgado, V. R., Megid, J., & Richtzenhain, L. J. (1997). A polymerase chain reaction for detection of brucella canis in vaginal swabs of naturally infected bitches. *Theriogenology*, *68,* 1260-1270. <https://doi.org/10.1016/j.theriogenology.2007.08.021>
