# Supplementary figures and images for: Epidemiological baseline of Brucella spp. in South African wildlife
Source: PLoS Negl Trop Dis. 2025 Dec 11;19(12):e0013754. doi: 10.1371/journal.pntd.0013754 (PMC12716795; doi:10.1371/journal.pntd.0013754)

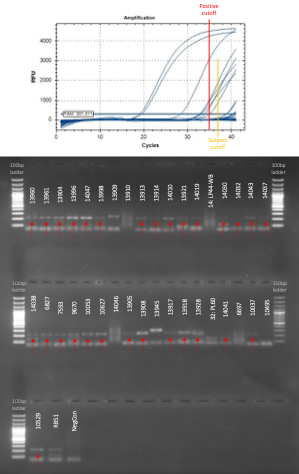

Supplement: S4 File — On top, typical amplification curves obtained with real-time PCR targeting the insertion sequence 711 (IS711), unique to Brucella genus. Positive cutoff indicated in red, “suspect” cutoff indicated in yellow. At the bottom, gel picture of 214 bp PCR products of the 16S-23S ITS region of Brucella spp., reloaded in series to verify correct band size. Red arrows indicate positive reactions. (PNG) [file pntd.0013754.s004.png]

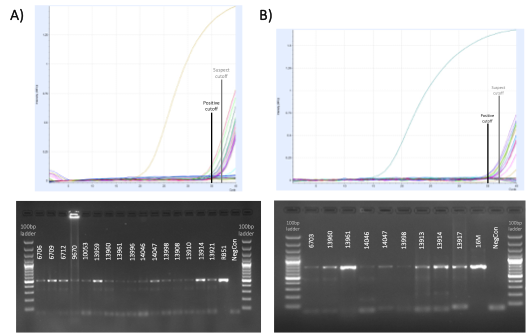

Supplement: S5 File — On top, typical amplification curves obtained with duplex real-time PCR. Positive cutoffs are indicated in black, “suspect” cutoffs indicated in grey. At the bottom, gel picture of some AMOS singleplex PCR products where B. abortus positive reactions are seen as 500 bp bands and B. melitensis as 700 bp bands. Products were here reloaded in series to double-check correct band size. (PNG) [file pntd.0013754.s005.png]
