# Supplementary material for: Epidemiological baseline of Brucella spp. in South African wildlife
Source: PLoS Negl Trop Dis. 2025 Dec 11;19(12):e0013754. doi: 10.1371/journal.pntd.0013754 (PMC12716795; doi:10.1371/journal.pntd.0013754)
Supplement: Attachment — Submitted filename: Responses_revs_251020.docx [file pntd.0013754.s008.docx]

Additional Editor Comments
The study is a valuable analysis of the presence of *Brucella* spp. in diverse animal wild life species in South Africa. However, the granularity of the study lacks critical information, including sampling strategy, period of data collection, health status of the animals or the origin of the different Brucella species identified in this work, among several missing parameters. The authors should precise these variables in order to truly allow to understand the persistence of *Brucella* in the studied ecosystem.

We thank the Editor for the constructive feedback and for recognizing the value of our study. We have now included comprehensive information to address the missing details highlighted. Specifically, the sampling strategy and period of data collection of the animals have been detailed in the revised Materials and Methods section under the “Sample area and collection” paragraph. Furthermore, we have compiled and provided all relevant metadata for the animals sampled as Supporting Files S1 and S2. These additions ensure transparency and reproducibility, providing the necessary context to better understand the persistence and epidemiological significance of *Brucella* spp. within the studied wildlife ecosystem.

Reviewers’ Comments

**Methods:**

**Reviewer #1**: The objectives of the study are clearly articulated.

The study design is appropriate to address the stated objectives.

However, the samples are not clearly described: the selection of the animals included in the study is not well documented. There is no supporting file listing the samples and their associated data. We don’t know when the samples were collected.

This lack of information makes it challenging to evaluate the study's methodology and reproducibility.

We thank the reviewer for this valuable comment. Comprehensive information on the animals sampled and their associated metadata has now been included as Supporting Files S1 and S2. Details regarding the timing of collection and sampling strategy have been added to the “sample area and collection” paragraph of the Materials and Methods section.

**Reviewer #2**: Which chemistry was used for real-time PCR experiments? SYBR Green/probes?. If SYBR green was used, melting curves would be necessary for specificity analysis.

We thank the reviewer for this insightful comment. As described in the original protocol (Hinić et al., 2008), the *Brucella* real-time PCR employed TaqMan hydrolysis probes.

Line 2111: All the sequences obtained in this study were deposited in GenBank database under the accession numbers: …

Please indicate

Accession numbers have been included as kindly indicated.

Supplementary figures would benefit from clarification. What do the red arrows indicate? It would be helpful to show in the graphic the size of the correct bands for B. abortus and B.melitensis

We thank the reviewer for this helpful suggestion. Correct band sizes are already detailed in the figure caption as follows: “*B. abortus*-positive reactions appear as 500 bp bands, and *B. melitensis*-positive reactions as 700 bp bands.” The purpose of the red arrows has now been clarified in the figure legend, as kindly suggested.

**Reviewer #3**: The authors have carried out extensive sampling work on numerous large wild animals. The article is clear and understandable.

This article is the result of international collaboration. The subject is important for detection and identification, particularly in the context of veterinary and human diagnosis. Numerous publications such as Qureshi et al., 2023, Middlebrook et al., 2022, Gakuya et al., 2022 (cited by the authors) and Dadar et al., 2021 describe the presence of Brucella spp. in wildlife and livestock in sub-Saharan Africa.

The originality of this article lies in the fact that it describes for the first time a very detailed epidemiology of the Brucella genus based on samples taken from more than 500 wild mammals, representing more than 20 different species, in protected areas in South Africa.

In the introduction, could you specify that Brucella is a highly pathogenic bacterium. Samples and isolates must be handled in a biosafety level 3 laboratory in order to protect the handler.

We thank the reviewer for this helpful remark. A statement specifying that *Brucella* is a highly pathogenic bacterium requiring handling in biosafety level 3 facilities has been added to the Introduction section.

Lines 39 to 41: Could you please specify the origin of the different Brucella species? For example, B. inopinata was isolated from human samples, B. microti was isolated from voles, and B. ceti was isolated from aquatic animals.

Additional details regarding the origin of the different *Brucella* species have been added as kindly suggested.

You mention the genus Ochrobactrum. Could you clarify that since 2020 (Hördt et al.), certain species of Ochrobactrum (O.) have become Brucella, such as O. anthropi, etc.

A note on the recent reclassification of certain *Ochrobactrum* species as *Brucella* has been added to the text as kindly suggested.

Line 138: it is noted ‘A total of 588 wild animals belonging to 22 species were sampled ...’. Table 1 lists 580 animals belonging to 23 species. Could you please correct this information ?

We thank the reviewer for highlighting this inconsistency. The discrepancy resulted from an earlier version of the dataset that included only animals sampled for molecular analyses. The final dataset includes all animals sampled for either molecular or serological analyses, totaling 722 animals representing 23 species. The text and Table 1 have been revised accordingly. Further information on the animal samples are provided in supporting files S1 and S2.

Line 212: It would appear that the number of sequences aligned with the MUSCLE algorithm is missing.

The number of sequences aligned with the MUSCLE algorithm has been added as kindly suggested.

Line 220 : The bibliographic reference for Farrell's medium preparation is missing.

The missing bibliographic reference for Farrell’s medium has been included as suggested.

The real-time PCR protocols are clear and combine the various PCRs needed to detect and identify the Brucella genus and its different species.

However, for the ‘Brucella spp. IS711 real-time PCR’ protocol, “BHQ1” is listed for the 3'Quencher, whereas in the article, the 3'Quencher is ‘TAMRA’. Could you please correct this ?

We thank the reviewer for noting this inconsistency. The correct 3′ quencher used in the *Brucella* spp. IS711 real-time PCR assay was BHQ1, not TAMRA. This adjustment from the original protocol was implemented to reduce non-specific amplification. The manuscript has been corrected accordingly, and the methodological revision is highlighted in the Materials and methods section (Lines 324–325).

In the reference to Keid et al., the year is listed as 1997, whereas the article was published in 2007. Could you please correct the date in the text of the article, in the bibliographical references and in the ‘Brucella spp. ITS Touchdown cPCR’ protocol ?

The reference to Keid et al. has been corrected from 1997 to 2007 in the text, reference list, and *Brucella* spp. ITS Touchdown cPCR protocol, as kindly indicated.

**Results**

**Reviewer #1**: The results are clearly presented. The figures and tables are clear.

However, the serological prevalence must be checked as it is 30/577 (line 258), 30/580 in table 1 and I count 27/577.

We thank the reviewer for drawing attention to this inconsistency. The correct number is indeed 27/577, and this has been corrected in both the text and Table 1 accordingly.

**Reviewer #2**: (No Response)

**Reviewer #3**: Line 258 : It is noted 30/580 in Table 1. Could you correct either of the values?

Line 263 : The values 30/580 are noted in the text and in Table 1. Are these the correct values ?

We thank the reviewer for highlighting this inconsistency. The correct number is 27/577, and all corresponding values have been corrected in the manuscript to ensure consistency.

Line 267 : « A total of 25/53 (47%) animals from LWR tested positive ». It should be noted that the small number of animals for certain species should be taken into account, with only one animal tested for African buffalo (1/1=100%), African elephant and cheetah (1/3=33%).

We thank the reviewer for this valuable observation. The limitations associated with small sample sizes for certain species are now explicitly acknowledged in the final part of the Discussion section.

The tables help to understand the text. However, a map of South Africa showing the locations of the various animal parks would be greatly appreciated by readers. On this map, you could also show where the largest number of Brucella spp. positive animals are located.

We thank the reviewer for this suggestion. However, we believe that including an additional figure showing sample locations stratified by molecular and serological results would be redundant, as Table 1 already reports detailed results by animal species and sampling area.

Figures S3 and S4 lack sharpness. Could you please improve the sharpness of the photos ?

We thank the reviewer for the observation. Figures S3 and S4 have now been resubmitted as high-resolution TIFF images to improve visual clarity.

**Conclusions:**

**Reviewer #1**: The conclusions are well supported by the data.

The limitations of analysis are clearly described.

The authors discuss well. However, without information on when the samples were collected, it is challenging to analyze the long-term persistence of Brucella in the animals over the years.

We thank the reviewer for this important comment. Detailed information on the animals sampled, including collection dates and associated metadata, has now been added as Supporting Files S1 and S2.

**Reviewer #2**: (No Response)

**Reviewer #3**: In the discussion, the reader clearly understands the fundamental role of monitoring Brucella spp. from asymptomatic wildlife and the role of climate change.

The authors show that certain animals contribute to the dynamics of pathogen transmission without being carriers of these pathogens.

The authors also mention the limitations of their study, in particular the viable isolation of Brucella from environmental or veterinary samples.

Among all the animals you studied, could you please clearly specify the number of healthy carriers and the number of animals showing symptoms ?

We thank the reviewer for this helpful comment. All animals included in this study were clinically healthy at the time of sampling, with no visible signs consistent with brucellosis (e.g., orchitis, arthritis, abortion). This information has now been explicitly stated in the Materials and Methods section (Lines 334–335).

Lines 326 to 327 : Specify the number of animals tested for each positive result (53% (9/17), 100% (4/4), 100% (3/3).

The number of animals tested for each positive result has been specified in the revised manuscript as kindly indicated.

Line 335 : ‘the circualtion’ should be corrected to ‘the circulation’

Corrected as kindly indicated.

**Editorial and Data Presentation Modifications**

**Reviewer #1**: Abstract

Line 25: Please, remove « was successfully » to make an understandable sentence or rewrite the sentence.

Corrected as kindly indicated.

Introduction

Lines 78 to 82: This sentence is too long. Please, cut it and indicate the reference number of Sambu et al. (2021) from the list of references. I think it is the 38th.

Lines 86 to 92: This sentence is too long. Please, cut it and indicate the reference number of Gakuya et al. (2022) from the list of references. I think it is the 32nd.

The sentences have been shortened as kindly suggested. As the journal follows the APA citation style, numerical references are not used in the main text.

Line 92: indicate the reference number of Katani et al. (2021) from the list of references. I think it is the 39th.

The citation style is APA. Therefore, numerical referencing is not applied in the manuscript.

Line 115. Please, insert a short sentence to explain who conceived and designed the study.

Thank you for the comment. This information is already included under the **CRediT taxonomy** section of the Author Contributions.

Materials and methods

Sample area and collection.

Line 126: Please remove “Fig.1” as this figure does not describe the parks.

Corrected as kindly indicated.

Line 138: the animals were sampled opportunistically. Could you precise the period of collect (several years?), the number of live and dead animals. How were the animals selected?

Additional information have been included in the “sample area and collection” paragraph as kindly indicated.

Line 147 to 153: Where are these supplementary data? Please provide a file with these data.

Included as kindly suggested.

Serological testing

Lines 174 to 176: RBT and ELISA detect the same antigen and display the same cross-reactions with other bacteria. So, using both RBT and ELISA does not improve the specificity, but probably improves the sensitivity of the serological testing. Could you change the sentence?

We thank the reviewer for this insightful observation. It is not solely a matter of antigen detection but also of conjugate variability. The iELISA multi-species conjugate reacts differently across host species, as reported by Ochai et al. (2023). For this reason, we retained the current wording, which reflects the rationale for combining RBT and iELISA. In our study, animals were classified positive only when positive with both tests, which increases the specificity of the detection. Additional limitations of serological testing are discussed in the final section of the Discussion section.

Line 176: Please indicate the reference numbers

Accession numbers included as kindly suggested.

Molecular testing

Line 190: Please indicate the reference numbers

Accession numbers included as kindly suggested.

DNA Sequencing

Line 212: the accession numbers are missing

Accession numbers included as kindly suggested.

MLVA

What does mean ARC-OVR?

Abbreviation has been written in full as kindly indicated.

Line 231: Please, correct retrieved from Liu et al (59) (supporting file S4)

Corrected as kindly indicated.

Line 233: Maybe you forgot “from” before Vergnaud et al.

Corrected as kindly indicated.

Results

Line 258: It seems that the serological prevalence is 27/577. It is not concordant with the results in line 258 (30/577) neither with the overall in table 1(30/580). Please, indicate the correct results.

We thank the reviewer for noting this inconsistency. The correct value is 27/577, which has been corrected throughout the text and Table 1.

Figure 2 legend. Line 288: write species instead of specues.

Corrected as kindly indicated.

Line 299: you could precise Fig. 3B instead of Fig. 3.

Corrected as kindly indicated.

Line 313: Please, make the raw data available for each animal in an additional supporting file: animal species, age group, park, sample type, date of sampling, condition dead or alive, serological and molecular tests.

The requested raw data have been made available as Supporting Files S1 and S2, as kindly suggested.

Lines 303 to 306: The MLVA-16 profiling of an unpure culture obtained from the spleen of a plains zebra was identical to that of the strain SAN94. This strain is listed in the supporting Excel file. However, I cannot find the other strains (Bru 0834, 0874….) in the file. Please, could you check or explain why?

The strains have been verified and corrected in the supporting file as kindly indicated.

Discussion

Line 344: African buffalo showed a high prevalence in GKNP. You assume that Brucella spp. is endemically established in GKNP. However, this high prevalence could result from an outbreak. Could you discuss it?

We thank the reviewer for this comment. The suggestion of endemicity in GKNP is not based only on prevalence data but primarily on phylogenomic analyses of *Brucella* isolates from African buffalo (Cossu et al., 2025). Further elaboration on this point would be redundant in this paper, as it is thoroughly addressed in that publication.

Line 357: A giraffe was confirmed positive by both serological and molecular methods. You were able to withdraw some blood and take a piece of liver on this animal? Was this giraffe sick?

The giraffe was sampled opportunistically following trophy hunting. Trophy hunting in Timbavati Private Reserve is conducted sustainably for population control and serves as an important source of funding for conservation programs. The authors do not deem necessary to specify this information in the main manuscript.

Line 392: remove “by”

Corrected as kindly indicated.

Line 419: Replace out ability by our ability.

Corrected as kindly indicated.

Supporting files

The supporting Information S2 is an xlsx file and correspond to the S4 file listed line 477.

Corrected as kindly indicated.

Please check the name of the files.

File names have been revised as kindly suggested.

Add a supporting file with the samples and their associated data.

Included as kindly suggested.

**Reviewer #2**: Line 16: Organ and serum samples from (n=588, animals representing 23 species) vs Line 138: A total of 588 wild animals belonging to 22 species

Please homogenize

Corrected as kindly indicated.

Line 287. Figure 2. Neighbour-joining phylogenetic tree displaying the evolutionary relationships of Brucella/Ochrobactrum species

Corrected as kindly indicated.

Line 336 Furthermore the findings suggest the circulation of brucellosis within specific South African wildlife populations, highlighting the limitations of relying solely on serological surveillance

Circulation

Corrected as kindly indicated.

**Reviewer #3**: (No Response)

**Summary and General Comments**

**Reviewer #1**: This study is notable for demonstrating the high prevalence of Brucella in African buffalo within the vast Kruger National Park, and for highlighting the superior sensitivity of PCR over serological tests in detecting Brucella infections in animals.

However, I have a significant concern regarding this manuscript. The selection of the animals included in the study is not well documented. There is no supporting file listing the samples and their associated data. We don’t know when the samples were collected.

This lack of information makes it challenging to evaluate the study's methodology and the conclusions.

We thank the reviewer for the constructive comments. The criteria and rationale for animal selection have now been comprehensively detailed in the “Sample area and collection” section. In addition, the time of sample collection has been explicitly indicated both in the revised text and in the raw data provided as Supporting Files S1 and S2. These files now include complete information on each sampled animal, encompassing species, location, sample type and collection date.

**Reviewer #2**: I read with interest the manuscript by Cossu et al. The ecological role of wild animal species in the sylvatic cycle of Brucella spp. remains unclear and needs further investigation. In the present study, the authors analyzed brucellosis status of wild animals from five of South Africa's nine provinces (Limpopo, Mpumalanga, Northern Cape, Western Cape, and Eastern Cape), providing a broad perspective on the brucellosis status in wildlife (a total of 588 wild animals belonging to 22 species). Diagnostic procedures combined serology (rose bengal test confirmed by indirect ELISA) and PCR-based assays. The paper is well written and interesting. Co-infections with B. abortus and B. melitensis were identified. Serological prevalence was low at 5% (30/580) while molecular prevalence was notably higher at 12% (70/585), suggesting that PCR may be more sensitive or capable of detecting infection in the absence of positive serological results. The present manuscript underscores the importance of combining molecular and serological tools for accurate detection and surveillance, highlighting the limitations of relying solely on serological surveillance.

We thank the reviewer for the encouraging and constructive evaluation. We appreciate the recognition of the study’s contribution to understanding *Brucella* dynamics in South African wildlife and agree that the complementary use of molecular and serological methods strengthens diagnostic accuracy and epidemiological interpretation.

**Reviewer #3**: (No Response)
