# Supplementary material for: Epidemiological baseline of Brucella spp. in South African wildlife
Source: PLoS Negl Trop Dis. 2025 Dec 11;19(12):e0013754. doi: 10.1371/journal.pntd.0013754 (PMC12716795; doi:10.1371/journal.pntd.0013754)
Supplement: Attachment — Submitted filename: Responses_revs_251101.docx [file pntd.0013754.s009.docx]

Additional Editor Comments

As suggested by Reviewer 3, please include a figure showing sample locations and number of animal studies on a map of South Africa, which would allow to better illustrate the study

We thank the editor for the opportunity to further improve the manuscript. In response of the requested modifications, we have added a new figure (Figure 2) illustrating the geographical distribution of sampled animal locations across South Africa, stratified by test results from serological (Fig. 2A) and molecular (Fig. 2B) analyses. This figure provides a visual complement to Table 1 by depicting both the sampling locations and relative distribution of test outcomes. All other figure numbers have been updated accordingly

## Reviewers' comments

**Methods**

Reviewer #1: The objectives are clearly articulated.
The study design is appropriate
The population is clearly described

We thank the reviewer for the positive feedback.

Reviewer #2: The revised version of the manuscript shows significant improvement and adequately addresses the reviewers´ comments.

We thank the reviewer for the positive feedback.

Reviewer #3: (No Response)

**Results**

Reviewer #1: The results are clear and completely presented.

We thank the reviewer for the positive feedback.

Reviewer #2: The revised version of the manuscript shows significant improvement and adequately addresses the reviewers´ comments.

We thank the reviewer for the positive feedback.

Reviewer #3: I consider the authors' answers to all questions to be well documented, however, the addition of a figure showing sample locations and number of animals studies on a map of South Africa is still missing to illustrate Table 1.
For instance see Figure 1 in the article by Lane et al., 2014
<https://pmc.ncbi.nlm.nih.gov/articles/PMC4159300/pdf/pone.0107038.pdf>

We thank the reviewer for the constructive comment. In response, we have added a new figure (Figure 2) illustrating the geographical distribution of sampled animal locations across South Africa, stratified by test results from serological (Fig. 2A) and molecular (Fig. 2B) analyses. This figure provides a visual complement to Table 1 by depicting both the sampling locations and relative distribution of test outcomes. All other figure numbers have been updated accordingly.

**Conclusions**
Reviewer #1: No comment

Reviewer #2: The revised version of the manuscript shows significant improvement and adequately addresses the reviewers´ comments.

We thank the reviewer for the positive feedback.

Reviewer #3: (No Response)

**Editorial and Data Presentation Modifications?**
Reviewer #1: no comment

Reviewer #2: The revised version of the manuscript shows significant improvement and adequately addresses the reviewers´ comments.

We thank the reviewer for the positive feedback.

Reviewer #3: (No Response)

**Summary and General Comments**
Reviewer #1: the manuscript has been improved so I have no more concern about it.

We thank the reviewer for the positive feedback.

Reviewer #2: The revised version of the manuscript shows significant improvement and adequately addresses the reviewers´ comments.

We thank the reviewer for the positive feedback.

Reviewer #3: (No Response)
